# Comprehensive diagnosis of advanced-stage breast cancer: exploring detection methods, molecular subtypes, and demographic influences – A cross-sectional study

**DOI:** 10.1016/j.clinsp.2024.100510

**Published:** 2024-10-15

**Authors:** Fernando Wladimir Silva Rivas, Rodrigo Gonçalves, Bruna Salani Mota, Isabel Cristina Esposito Sorpreso, Tatiana Natasha Toporcov, José Roberto Filassi, Edia di Tullio Lopes, Laura Raíssa Schio, Yann-Luc Patrick Comtesse, Edmund Chada Baracat, José Maria Soares Júnior

**Affiliations:** a Disciplina de Ginecologia, Departamento de Obstetrícia e Ginecologia, Hospital das Clínicas, Faculdade de Medicina da Universidade de São Paulo, São Paulo, SP, Brazil; b Setor de Mastologia da Disciplina de Ginecologia, Instituto de Câncer do Estado de São Paulo, Hospital das Clínicas, Faculdade de Medicina da Universidade de São Paulo, São Paulo, SP, Brazil; c Departamento de Epidemiologia, Faculdade de Saúde Pública, Universidade de São Paulo, São Paulo, SP, Brazil; d Registro Hospitalar de Câncer, Serviço de Arquivo Médico, Instituto de Câncer do Estado de São Paulo, São Paulo, SP, Brazil

**Keywords:** Breast Cancer, Screening, Advanced-stage, Social determinants of health, Brazil

## Abstract

**Background:**

Brazil faces notable Breast Cancer (BC) mortality despite lower incidence rates versus developed countries. Despite guidelines from medical societies, Brazilian public policy recommends biennial mammographic screening for women aged 50 to 69. This study investigates sociodemographic and clinical factors related to BC detection methods and clinical stage at diagnosis.

**Methods:**

The authors conducted a cross-sectional study at a São Paulo tertiary hospital. Patients were divided into ‘symptomatic’ and ‘mammographic’ detection groups. Bivariate analyses by detection method and clinical stage compared groups' profiles in terms of sociodemographic and clinical characteristics. Poisson regression analyses assessed sociodemographic and molecular subtypes´ influence on “mammographic detection” prevalence and “advanced-stage BC”, reporting prevalence ratios and 95 % Confidence Intervals.

**Results:**

The authors studied 1,536 BC patients admitted from January 2016 to December 2017. The “mammographic detection” group had a higher proportion of patients aged 50‒69 years (62.9 % vs. 44.1 %), white race (63.3 % vs. 51.6 %), Catholic religion (58.2 % vs. 51.1 %), and Luminal A subtype (25.2 % vs. 13.2 %) compared to the “symptomatic detection” group. Patients with early-stage disease were more likely to have higher education levels (8.1 % vs. 5.5 %) and be married (39.8 % vs. 46.6 %) compared to those with advanced-stage. Molecular subtypes were significantly associated with the detection method and stage. The prevalence of advanced-stage disease in "mammographic" (n=313) and "symptomatic" (n=1191) groups was 18.5 % and 55 %, respectively . Mammographic detection significantly reduced advanced-stage BC prevalence (PR = 0.40, 95 % CI 0.31‒0.51).

**Conclusion:**

Mammographic detection reduces advanced-stage breast cancer prevalence in Brazil, emphasizing the importance of regular screenings, especially among at-risk sociodemographic groups. Enhancing mammographic screening accessibility, lowering the starting age to 40, and extending coverage to include annual mammograms can significantly lower breast cancer mortality in Brazil, benefiting public health and patient outcomes.

## Highlights

• In Brazil, breast cancer leads to 94,728 new cases and 22,189 deaths annually.
• Early diagnosis and molecular profiles significantly impact breast cancer mortality.
• Mammographic detection associated with lower prevalence of advanced-stage cancer.
• Age, marital status, and race/color significantly influenced detection methods.
• Luminal A tumors had the highest detection rates; triple-negative the lowest.

## Introduction

Breast Cancer (BC) is a significant cause of mortality among women in Brazil. Data from 2022 indicate 94,728 new cases, resulting in 22,189 recorded deaths.^1^ Mortality related to BC is intricately linked to factors such as early-stage diagnosis, timely and adequate treatment, and the molecular profiles of the disease.^2^, ^3^, ^4^, ^5^ Molecular diagnosis of BC is considered crucial for improving outcomes.^4^^,^^6^ Molecular intrinsic subtypes determine treatment requirements and can be inferred by evaluating the expression of estrogen and progesterone receptors, HER2 protein, and cell proliferation measured by Ki-67.^7^ Clinical Stage (CS) at diagnosis plays a vital role in predicting outcomes, and mammographic screening remains the most effective tool in mitigating Advanced-stage BC (ABC) and reducing mortality.^8^

In Brazil, public policy endorses biennial mammographic screening for women aged 50‒69 years old.^8^ Recent recommendations from medical associations, however, advocate for annual mammographic screening for women aged 40‒74.^9^ Mammographic screening in Brazil is described as “opportunistic”.^10^, ^11^, ^12^, ^13^, ^14^, ^15^, ^16^ The program's coverage varies widely, ranging from slightly over 1 % in less developed states such as Amapá to 28.8 % in São Paulo, the most populated Brazilian state,^17^ in 2017. Despite initial increasing trends and stabilization in recent decades,^17^ social inequities remain significant barriers. Brazil's heterogeneous social and cultural background, including factors such as age, education, income, marital status, race/color, and religion, has been associated with non-adherence to BC mammographic screening,^14^^,^^18^, ^19^, ^20^, ^21^ ABC at diagnosis,^22^, ^23^, ^24^ delayed treatment,^9^^,^^25^^,^^26^ and prognosis.^2^^,^^27^, ^28^, ^29^, ^30^

This study aims to compare the sociodemographic characteristics and molecular subtypes of patients categorized by the BC detection method, estimating the prevalence of mammographic detection within these groups. Additionally, this study aims to compare the sociodemographic characteristics and molecular subtypes of patients according to the CS of the disease.

## Materials and methods

### Study design and data sources

This is a cross-sectional study designed to evaluate the impact of mammographic detection, clinical factors, and sociodemographic variables on BC mortality in a tertiary hospital in Brazil. Data were collected from the Hospital-based Cancer Registry (HCR) of the *Instituto do Câncer do Estado de São Paulo* (ICESP) and supplemented with patient-level data from Electronic Medical Records (EMR).

### Eligibility criteria

The authors included female patients aged 18 and above with a histologically confirmed diagnosis of BC at any stage, admitted between January 1, 2016, and December 31, 2017, and subsequently followed until February 2024. Eligible patients were those who consulted at the ICESP outpatient clinic without a prior BC diagnosis or with a BC diagnosis but had not yet initiated treatment. Patients with previous oncological diagnoses, new primary malignant neoplasms within five years following their BC diagnosis, duplicated records, or incomplete medical records were excluded.

### Study variables

The HCR provides standardized information on patients and diseases, including age at diagnosis, education level, and clinical staging using the TNM system. This system defines the size of the primary Tumor (cT), the extent of disease in regional lymph Nodes (cN), and the status of distant Metastasis (cM), as outlined in the AJCC Cancer Staging Manual, Seventh Edition.^31^ Clinical stages from 0 to IV were defined and grouped into “early-stage BC” (stages 0‒II) or “ABC” (stages III‒IV).

From the EMR, the authors extracted information on self-reported race, marital status, and religion, using categories defined by the Brazilian Institute of Geography and Statistics (IBGE). Additionally, the authors collected information about family history of cancer in first- and second-degree relatives. Based on initial assessments at the outpatient clinic, the authors defined the study groups. The BC detection method was classified as Symptomatic Detection (SD) for patients seeking medical attention after experiencing disease symptoms, or Mammographic Detection (MD) for patients consulting due to abnormal mammographic findings in the absence of symptoms or disease signs during the initial physical examination.

Information from immunohistochemical analyses of Estrogen Receptor (ER), Progesterone Receptor (PR), HER2 protein expression, and the Ki-67 score was also extracted. Molecular intrinsic subtypes were determined according to the 13th St. Gallen Consensus.^7^

### Statistical analysis

The authors performed bivariate analysis to compare the proportions of molecular subtypes and sociodemographic variables within the groups defined by detection method and CS at diagnosis. Absolute and relative frequencies were calculated for each category. Chi-Square tests were conducted to compare the proportions of each category within the present study groups.

To determine the prevalence of MD and ABC, the authors described the prevalence using absolute and relative frequencies for each category of independent variables. Poisson regression was employed for univariate and multivariate analyses in cross-sectional designs.^32^ Prevalence Ratios (PR), 95 % Confidence Intervals (95 % CI), and p-values were reported for each level of independent variables relative to a reference category. Small subgroups with missing data (n < 30) were excluded from the multivariate analysis.

All analyses were conducted using STATA 14 software and p-values were considered significant when p < 0.05. All eligible patients were included in a non-randomized manner and sample size and power analysis were not calculated. Obtaining informed consent from all subjects or their legal guardians was waived by the ethics committee. Ethical approvals were obtained from the ICESP Scientific Teaching and Research Commission, the ethics committee of the Department of Obstetrics and Gynecology at FMUSP, and the Ethics in Research Committee of USP-Hospital das Clínicas at the University of São Paulo School of Medicine, protocol number CAAE: 41107120.6.0000.0068. This study was performed in accordance with the Declaration of Helsinki. All procedures were performed according to ICESP´s breast cancer treatment guidelines and following the local regulations.

## Results

### Patient selection and characterization of study groups

**Figure 1:**
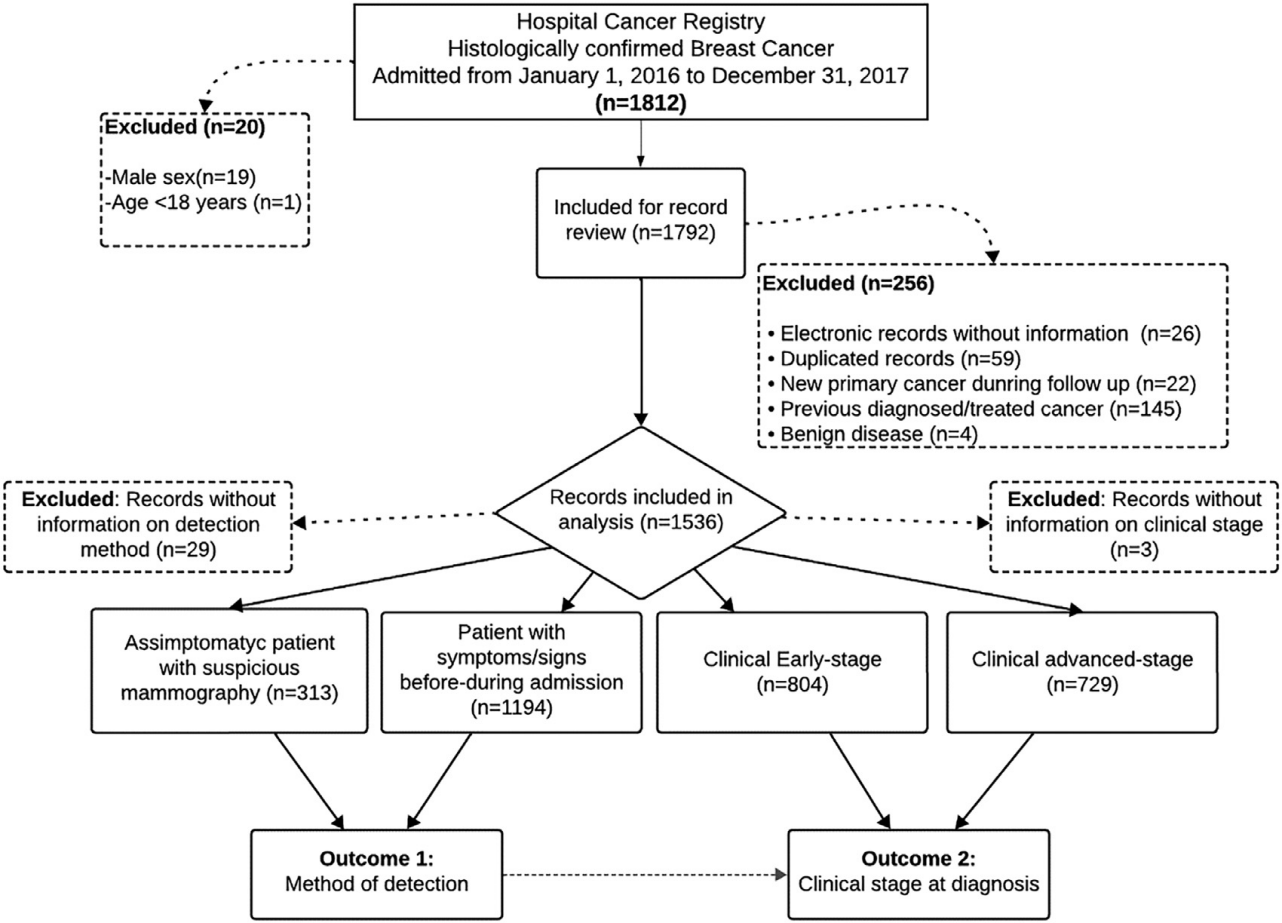
Patient selection algorithm and study design.

A total of 1,812 new cases were admitted to the Hospital-based Cancer Registry (HCR) under ICD10 code C50 from January 1, 2016, to December 31, 2017. All cases underwent diagnostic biopsies for histologic confirmation of BC. The authors excluded 19 male patients and 1 female patient below 18 years old. Thus, a total of 1,792 eligible records were examined, with 256 exclusions (reasons presented in Fig. 1). A sample of 1,536 records was included for subsequent statistical analyses.

Study groups were defined based on the method of BC detection: 313 asymptomatic patients (20.4 %) presented abnormal mammographic findings, 1,194 symptomatic patients (77.7 %) reported prior symptoms or had disease signs observed during the initial physical examination, and 29 patients (1.9 %) did not have a method of detection reported in the EMR.

At diagnosis, 804 patients (52.3 %) were classified in the ‘early-stage’ group, 729 patients (47.5 %) in the ABC group, and information on CS was missing for 3 patients (0.2 %) (Fig. 1).

### Patients’ characteristics by detection method

The MD and SD groups exhibited distinct profiles in age, marital status, race/color, religion, molecular subtype, and tumor and node stages (Table 1). There were no statistically significant differences between the study groups in proportions of education levels and family history of cancer. The MD group had a significantly higher proportion of patients aged 50‒69 years (62.9 % vs. 44.1 %) and 40‒49 years (24.4 % vs. 18.5 %), while fewer patients below 40 years were observed in the MD group (2.2 % vs. 15.8 %). Moreover, the MD group included more married individuals (49.5 % vs. 42.4 %), a higher percentage of individuals of white race/color (63.3 % vs. 51.3 %), and fewer individuals identifying as evangelical (24.9 % vs. 32.3 %) compared to the SD group.

**Table 1:** Sociodemographic and diagnostic characteristics by detection method and clinical stage at diagnosis.

	Method of detection (n = 1507)					Clinical stage at diagnosis (n = 1533)				
	Symptomatic (n = 1194)		Mammographic (n = 313)			Early-stage (n = 804)		Advanced stage (n = 729)		
	n	%	n	%	p-value	n	%	n	%	p-value
**Age group**					**<0.001**					**<0.001**
20‒39	189	15.8	7	2.2	**<0.001**	86	10.7	114	15.6	**0.004**
40‒49	291	24.4	58	18.5	**0.029**	185	23.0	168	23.1	0.987
50‒69	527	44.1	197	62.9	**<0.001**	418	52.0	321	44.0	**0.002**
70+	187	15.7	51	16.3	0.785	115	14.3	126	17.3	0.109
**Educational level**					0.712					0.196
Incomplete primary education	265	22.2	69	22.0	0.955	165	20.5	175	24.0	0.101
Complete primary education	131	11.0	31	9.9	0.587	86	10.7	80	11.0	0.861
High school	147	12.3	31	9.9	0.240	99	12.3	85	11.7	0.694
Superior	83	7.0	22	7.0	0.962	65	8.1	40	5.5	**0.044**
Not reported	568	47.6	160	51.1	0.264	389	48.4	349	47.9	0.842
**Marital status**					0.054					**0.003**
Married	506	42.4	155	49.5	**0.046**	382	47.5	287	39.4	**0.001**
Divorced	152	12.7	46	14.7	0.595	111	13.8	89	12.2	0.354
Widowed	339	28.4	72	23.0	0.113	196	24.4	225	30.9	**0.004**
Single	184	15.4	39	12.5	0.398	110	13.7	118	16.2	0.169
Not reported	13	1.1	1	0.3	0.184	5	0.6	10	1.4	0.136
**Race/color**					**0.004**					**0.007**
White	616	51.6	198	63.3	**0.001**	466	58.0	364	49.9	**0.002**
Brown	321	26.9	69	22.0	0.179	202	25.1	194	26.6	0.506
Black	72	6.0	9	2.9	0.084	40	5.0	42	5.8	0.494
Asian	7	0.6	2	0.6	0.911	4	0.5	5	0.7	0.630
Not reported	178	14.9	35	11.2	0.203	92	11.4	124	17.0	**0.002**
**Religion**					**0.032**					0.082
Catholic	610	51.1	182	58.2	0.076	436	54.2	369	50.6	0.157
Evangelical	386	32.3	78	24.9	**0.041**	232	28.9	240	32.9	0.085
Spiritism	49	4.1	20	6.4	0.110	44	5.5	25	3.4	0.054
None	49	4.1	9	2.9	0.599	33	4.1	26	3.6	0.585
Other*	40	3.4	13	4.2	0.464	27	3.4	26	3.6	0.824
Not reported	60	5.0	11	3.5	**0.005**	32	4.0	43	5.9	0.082
**Family history of cancer**					**0.379**					**0.005**
None	524	43.9	126	40.3	0.248	317	39.4	346	47.5	**0.002**
Breast/Ovaries	358	30.0	94	30.0	0.987	262	32.6	197	27.0	**0.018**
Other malign neoplasms	312	26.1	93	29.7	0.203	225	28.0	186	25.5	0.275
**Molecular subtype**					**<0.001**					**<0.001**
Luminal A	158	13.2	79	25.2	**<0.001**	183	22.8	56	7.7	**<0.001**
Luminal B (HER2 -)	528	44.2	121	38.7	0.136	320	39.8	338	46.4	**0.010**
Luminal B (HER2 +)	156	13.1	22	7.0	**0.013**	71	8.8	111	15.2	**<0.001**
Enriched HER2	95	8.0	17	5.4	0.225	40	5.0	73	10.0	**<0.001**
Triple negative	206	17.3	25	8.0	**<0.001**	106	13.2	131	18.0	**0.010**
HR+ unknown Her2/Ki67	36	3.0	42	13.4	**<0.001**	71	8.8	11	1.5	**<0.001**
Unknown	15	1.3	7	2.2	0.347	13	1.6	9	1.2	0.53
**Clinical Tumor Stage**					**<0.001**					
0	33	2.8	57	18.2	**<0.001**					
I	142	11.9	129	41.2	**<0.001**					
II	424	35.5	92	29.4	0.127					
III	254	21.3	16	5.1	**<0.001**					
IV	331	27.7	19	6.1	**<0.001**					
Unknown	10	0.8	0	0.0	0.237					
**Clinical Node Stage**					**<0.001**					
0	420	35.2	218	69.7	**<0.001**					
1	438	36.7	58	18.5	**<0.001**					
2	234	19.6	30	9.6	**<0.001**					
3	94	7.9	7	2.2	**0.002**					
Unknown	8	0.7	0	0.0	0.316					
**Method of detection**										**<0.001**
Symptomatic						536	66.7	655	89.9	**<0.001**
Mammographic						255	31.7	58	8.0	**<0.001**
Unknown						13	1.6	16	2.2	0.407

Tumor size was strongly associated with the detection method. The MD group showed a higher prevalence of Tis (18.2 % vs. 2.8 %) and cT1 tumors (41.2 % vs. 11.9 %), with a non-significant difference in the proportion of cT2 tumors. Furthermore, significantly lower proportions of cT3 (5.1 % vs. 21.3 %) and cT4 tumors (6.1 % vs. 27.7 %) were observed in the MD group. Differences in lymph node stage were also significant, with the MD group having higher proportions of cN0 (69.7 % vs. 35.2 %) and lower proportions of cN1 (18.5 % vs. 36.7 %), cN2 (9.6 % vs. 19.6 %), and cN3 (2.2 % vs. 7.9 %) compared to the SD group.

### Patients’ characteristics by stage at diagnosis

The groups with early-stage and ABC showed statistically significant differences in age, educational levels, marital status, race/color, family history of cancer, method of detection, and molecular subtypes (Table 1). The ABC group at diagnosis included a higher proportion of patients aged 20‒39 years (15.6 % vs. 10.7 %) and a lower proportion of patients aged 50‒69 years (44 % vs. 52 %) compared to the early-stage group. Additionally, a smaller proportion of individuals with higher education was observed in the advanced-stage group (5.5 % vs. 8.1 %).

Marital status differences were notable, with a lower proportion of married women in the advanced-stage group (39.4 % vs. 47.5 %) and a higher proportion of widowed women (30.9 % vs. 24.4 %). Furthermore, the advanced-stage group had fewer white women (49.9 % vs. 58 %) and a higher proportion of individuals with unreported race/color compared to the early-stage group (17 % vs. 11.4 %). Regarding family history, the advanced-stage group had a higher proportion of patients without a family history of cancer (47.5 % vs. 39.4 %) and a lower proportion with a family history of breast/ovarian cancer (27 % vs. 32.6 %) compared to the early-stage group.

Molecular subtypes also differed significantly between groups. The ABC group exhibited fewer luminal A tumors (7.7 % vs. 22.8 %) and a lower proportion of hormone receptor-positive tumors without information on HER2 status or Ki-67 (1.5 % vs. 8.8 %) compared to the early-stage group. Conversely, the ABC group showed higher proportions of HER2-negative Luminal B (46.4 % vs. 39.8 %), HER2-positive Luminal B (15.2 % vs. 8.8 %), Enriched HER2 (10 % vs. 5 %), and triple-negative tumors (18 % vs. 13.2 %).

Finally, the early-stage BC group had a lower proportion of patients presenting symptoms (66.7 % vs. 89.9 %) and a higher proportion of MD (31.7 % vs. 8 %) compared to the ABC group. The proportion of unknown methods of detection was balanced between the CS groups.

### Prevalence of mammographic detection by sociodemographic characteristics and molecular subtypes

Poisson univariate regression revealed significant differences in the prevalence of mammographic detection across age, marital status, race/color, religion, and molecular subtypes (Table 2). Patients aged 50‒69 years showed the highest prevalence of mammographic detection at 27.7 %. In contrast, patients aged 20‒39 had a significantly lower prevalence of 3.6 % (PR = 0.13, 95 % CI 0.06‒0.27), and those aged 40‒49 had a prevalence of 16.6 % (PR = 0.61, 95 % CI 0.47‒0.79). The prevalence difference for patients over 70 years old (21.4 %) was non-significant. Marital status also influenced MD prevalence, with married patients (reference group) showing a prevalence of 23.5 %. Widowed women had a reduced prevalence of 17.5 % (PR = 0.75, 95 % CI 0.58‒0.96).

**Table 2:** Prevalence of mammographic detection of breast cancer according to sociodemographic characteristics and molecular subtype, unadjusted coefficients and multivariate Poisson model.

Prevalence of mammographic detection (n = 1507)			Poisson Regression							
			Unadjusted prevalence ratios				Multivariate model (n = 1471)			
	n	%	PR	p-value	95 % CI		PR	p-value	95 % CI	
**Age group**										
50‒69	197/724	27.2	(1 ref.)				(1 ref.)			
20‒39	7/196	3.6	0.13	**<0.001**	0.06	0.27	0.16	**<0.001**	0.08	0.33
40‒49	58/349	16.6	0.61	**<0.001**	0.47	0.79	0.64	**<0.001**	0.50	0.83
70+	51/238	21.4	0.79	0.084	0.60	1.03	0.91	0.535	0.69	1.22
**Educational level**										
Incomplete primary education	69/334	20.7	(1 ref.)				(1 ref.)			
Complete primary education	31/162	19.1	0.93	0.693	0.63	1.35	0.93	0.708	0.64	1.35
High school	31/178	17.4	0.84	0.382	0.57	1.24	1.02	0.923	0.71	1.46
Superior	22/105	21.0	1.01	0.948	0.66	1.55	1.10	0.644	0.72	1.69
Not reported	160/728	22.0	1.06	0.629	0.83	1.37	1.15	0.258	0.90	1.47
**Marital status**										
Married	155/661	23.5	(1 ref.)				(1 ref.)			
Divorced	46/198	23.2	0.99	0.950	0.74	1.32	0.96	0.756	0.72	1.27
Widowed	72/411	17.5	0.75	**0.023**	0.58	0.96	0.82	0.116	0.65	1.05
Single	39/223	17.5	0.75	0.070	0.54	1.02	0.68	**0.019**	0.49	0.94
Not reported	1/14	7.1	0.30	0.219	0.05	2.03	**			
**Race/color**										
White	198/814	24.3	(1 ref.)				(1 ref.)			
Brown/Black	78/471	16.6	0.68	**0.001**	0.54	0.86	0.84	0.163	0.66	1.07
Not reported	37/222	16.7	0.69	**0.020**	0.50	0.94	0.82	0.219	0.60	1.12
**Religion**										
Catholic	182/792	23.0	(1 ref.)				(1 ref.)			
Evangelical	78/464	16.8	0.73	**0.010**	0.58	0.93	0.84	0.143	0.67	1.06
Spiritism	20/69	29.0	1.26	0.244	0.85	1.86	1.17	0.417	0.81	1.69
None	9/58	15.5	0.68	0.210	0.37	1.25	0.76	0.368	0.42	1.39
Other*	13/53	24.5	1.07	0.794	0.65	1.74	1.11	0.680	0.69	1.78
Not reported	11/71	15.5	0.67	0.166	0.39	1.18	0.82	0.493	0.45	1.46
**Family history of cancer**										
None	126/650	19.4	(1 ref.)				(1 ref.)			
Breast/Ovaries	94/452	20.8	1.07	0.564	0.85	1.36	1.04	0.715	0.83	1.32
Other malign neoplasms	93/405	23.0	1.18	0.162	0.93	1.50	1.17	0.182	0.93	1.47
**Molecular subtype**										
Luminal A	79/237	33.3	(1 ref.)				(1 ref.)			
Luminal B (HER2-)	121/649	18.6	0.56	**<0.001**	0.44	0.71	0.62	**<0.001**	0.49	0.79
Luminal B (HER2+)	22/178	12.4	0.37	**<0.001**	0.24	0.57	0.43	**<0.001**	0.28	0.66
Enriched HER2	17/112	15.2	0.46	**<0.001**	0.28	0.73	0.49	**0.002**	0.30	0.77
Triple negative	25/231	10.8	0.32	**<0.001**	0.22	0.49	0.39	**<0.001**	0.26	0.58
HR+ unknown Her2/Ki67	42/78	53.9	1.62	**0.001**	1.23	2.12	1.68	**<0.001**	1.27	2.22
Unknown	7/22	31.8	0.95	0.886	0.50	1.81	**			

Regarding race/color, white women exhibited a prevalence of 24.3 %, while black/brown women had a significantly lower prevalence of 16.6 % (PR = 0.68, 95 % CI 0.54‒0.86). Unreported race/color showed a prevalence of 16.7 % (PR = 0.69, 95 % CI 0.50‒0.94).

In terms of religion, Catholic patients (reference group) had a prevalence of 23 %, whereas Evangelic patients showed a prevalence of 16.8 % (PR = 0.73, 95 % CI 0.58‒0.93).

Significant differences in the prevalence of MD were also observed among molecular subtypes. Luminal A tumors had the highest prevalence at 33.3 %, whereas Luminal B (HER2 negative) had a prevalence of 18.6 % (PR = 0.56, 95 % CI 0.44‒0.72), Luminal B (HER2 positive) had 12.4 % (PR = 0.37, 95 % CI 0.27‒0.57), enriched HER2 subtype had 15.2 % (PR = 0.46, 95 % CI 0.28‒0.73), and triple-negative tumors showed 10.8 % (PR = 0.32, 95 % CI 0.22‒0.49). Patients with hormone receptor-positive tumors without information on HER2 had a prevalence of 53.9 %, significantly higher than the reference group (PR = 1.62, 95 % CI 1.23‒2.12). No statistical differences were found in the prevalence of MD between groups of educational levels and family history of cancer.

Multivariate regression analysis further confirmed that age 20‒39 (PR = 0.16, 95 % CI 0.08‒0.33) and 40‒49 (PR = 0.64, 95 % CI 0.50‒0.83) maintained lower prevalence rates compared to age 50‒69. Additionally, single women presented significantly lower prevalence rates than married women (PR = 0.68, 95 % CI 0.49‒0.94).

Compared to Luminal A tumors, other molecular subtypes showed significantly lower prevalence rates of MD: HER2 negative Luminal B (PR = 0.56, 95 % CI 0.49‒0.79), HER2 positive Luminal B (PR = 0.43, 95 % CI 0.28‒0.66), enriched HER2 subtype (PR = 0.49, 95 % CI 0.30‒0.77), and triple-negative (PR = 0.39, 95 % CI 0.26‒0.58). Hormone receptor-positive tumors without information on HER2 exhibited a higher prevalence compared to Luminal A tumors (PR = 1.68, 95 % CI 1.27‒2.22).

Multivariate regression showed non-significant differences in race/color, religion, family history of cancer, and education levels.

### Prevalence of advanced-stage breast cancer at diagnosis by sociodemographic characteristics and molecular subtypes

Univariate Poisson analysis showed significant differences in the prevalence of ABC at diagnosis across various patient characteristics, including age, marital status, race/color, family history of cancer, detection method, and molecular subtypes (Table 3).

**Table 3:** Prevalence of advanced clinical stage at diagnosis according to sociodemographic characteristics and molecular subtype, unadjusted coefficients and multivariate Poisson models.

Prevalence of advanced stage at diagnosis (n = 1533)			Poisson Regression							
				Unadjusted prevalence ratios			Multivariate model (n = 1468^a^)			
	n	%	PR	p-value	95 % CI		PR	p-value	95 % CI	
**Age group**										
50‒69	321/739	43.4	(1 ref.)				(1 ref.)			
20‒39	11//200	57.0	1.31	**<0.001**	1.13	1.52	1.08	0.324	0.93	1.25
40‒49	168/353	47.6	1.10	0.191	0.96	1.26	1.02	0.719	0.90	1.17
70+	126/241	52.3	1.20	**0.013**	1.04	1.39	1.17	0.041	1.01	1.36
**Educational level**										
Incomplete primary education	175/340	51.5	(1 ref.)				(1 ref.)			
Complete primary education	80/166	48.2	0.94	0.494	0.78	1.13	0.93	0.424	0.78	1.11
High school	85/184	46.2	0.90	0.257	0.74	1.08	0.91	0.338	0.76	1.10
Superior	40/105	38.1	0.74	**0.026**	0.57	0.96	0.80	0.089	0.62	1.03
Not reported	349/738	47.3	0.92	0.196	0.81	1.04	0.95	0.450	0.85	1.08
**Marital status**										
Married	287/669	42.9	(1 ref.)				(1 ref.)			
Divorced	89/200	44.5	1.04	0.686	0.87	1.24	0.93	0.424	0.78	1.11
Widowed	225/421	53.4	1.25	**0.001**	1.10	1.41	0.91	0.338	0.76	1.10
Single	118/228	51.8	1.21	**0.016**	1.04	1.41	0.80	0.089	0.62	1.03
Not reported	10/15	66.7	1.55	**0.019**	1.08	2.25	0.95	0.450	0.85	1.08
**Race/color**										
White	364/830	43.9	(1 ref.)				(1 ref.)			
Brown/Black	236/478	49.4	1.13	0.051	1.00	1.27	0.97	0.660	0.86	1.10
Not reported	129/225	57.3	1.31	**<0.001**	1.14	1.50	1.18	0.013	1.04	1.34
**Religion**										
Catholic	369/805	45.8	(1 ref.)				(1 ref.)			
Evangelical	240/472	50.9	1.11	0.080	0.99	1.25	1.07	0.275	0.95	1.19
Spiritism	25/69	36.2	0.79	0.152	0.57	1.09	0.89	0.436	0.66	1.19
None	26/59	44.1	0.96	0.795	0.71	1.29	0.96	0.799	0.71	1.30
Other^a^	26/53	49.1	1.07	0.640	0.81	1.42	1.13	0.405	0.85	1.49
Not reported	43/75	57.3	1.25	0.036	1.01	1.54	1.21	0.094	0.97	1.52
**Family history of cancer**										
None	346/663	52.2	(1 ref.)				(1 ref.)			
Breast/Ovaries	197/459	42.9	0.82	**0.003**	0.72	0.93	0.84	0.004	0.74	0.94
Other malign neoplasms	186/411	45.3	0.87	**0.030**	0.76	0.99	0.89	0.061	0.79	1.01
**Molecular subtype**										
Luminal A	56/239	23.4	(1 ref.)				(1 ref.)			
Luminal B (HER2-)	338/658	51.4	2.19	**<0.001**	1.72	2.79	1.97	0.000	1.55	2.50
Luminal B (HER2+)	111/182	61.0	2.60	**<0.001**	2.01	3.37	2.23	0.000	1.72	2.90
Enriched HER2	73/113	64.6	2.76	**<0.001**	2.11	3.60	2.45	0.000	1.87	3.19
Triple negative	131/237	55.3	2.36	**<0.001**	1.83	3.05	2.06	0.000	1.60	2.66
HR+ unknown Her2/Ki67	11/82	0.4	0.57	0.067	0.32	1.04	0.71	0.244	0.39	1.27
Unknown	9/22	40.9	1.75	**0.048**	1.01	3.03				
**Method of detection**										
Symptomatic	655/1191	55.0	(1 ref.)				(1 ref.)			
Mammographic	58/313	18.5	0.34	**<0.001**	0.27	0.43	0.40	0.000	0.31	0.51
Unknown	16/29	55.2	1.00	0.985	0.72	1.40				

a Cases labeled as “unknown” molecular subtype (n = 22), “unknown” method of detection (n = 29), “not reported” marital status (n = 15) was excluded of multivariate model, totalizing 65 excluded observations, since one case had no information on method of detection and marital status. The resulting reduction in sample size was less than 5 % of the data set. HR, Hormonal Receptors; HER2, Human Epidermal Growth Factor Receptor 2; 95 % CI, Confidence interval 95 %; PR, Prevalence ratio.

The reference age group, women with 50‒69 years, had a prevalence of ABC of 43.4 %. Patients aged 20‒39 had a prevalence of 57 % (PR = 1.31, 95 % CI 1.13‒1.52), while those over 70 years had a prevalence of 52.3 % (PR = 1.20, 95 % CI 1.04‒1.39). Education level also influenced prevalence, with the reference group (incomplete primary education) showing a 51.5 % prevalence of ABC. Women with higher education had a lower prevalence of 38.1 % (PR = 0.74, 95 % CI 0.57‒0.96). Race/color was another significant factor. The prevalence among white women was 43.9 %, while individuals with unreported race/color had a prevalence of 57.3 % (PR = 1.31, 95 % CI 1.14‒1.50). Family history of cancer revealed that individuals without a family history had a 52.2 % prevalence of ABC. Patients with a family history of breast/ovarian cancer had a lower prevalence of 42.9 % (PR = 0.82, 95 % CI 0.72‒0.93), and those reporting any other cancer had a prevalence of 45.3 % (PR = 0.87, 95 % CI 0.76‒0.99).

Married women had a 42.9 % prevalence of ABC. In contrast, widowed women had a prevalence of 53.4 % (PR = 1.25, 95 % CI 1.10‒1.41), single women had a prevalence of 51.8 % (PR = 1.21, 95 % CI 1.04‒1.41), and those with unreported marital status had a prevalence of 66.7 % (PR = 1.55, 95 % CI 1.08‒2.25).

Molecular subtypes also showed significant differences. Compared to Luminal A with a 23.4 % prevalence, HER2 negative Luminal B had a prevalence of 51.4 % (PR = 2.19, 95 % CI 1.72‒2.79), HER2 positive Luminal B had 61 % (PR = 2.60, 95 % CI 2.01‒3.37), enriched HER2 had 64.6 % (PR = 2.76, 95 % CI 2.11‒3.60), and triple-negative had 55.3 % (PR = 2.36, 95 % CI 1.83‒3.05). The unknown subtype showed a prevalence of 40.9 % (PR = 1.75, 95 % CI 1.01‒3.03).

The detection method was strongly associated with the stage at diagnosis. Symptomatic patients had a prevalence of 55 %, while mammographic detected cases had a prevalence of 18.5 % (PR = 0.34, 95 % CI 0.27‒0.43).

Multivariate regression analysis confirmed that age, marital status, race/color, family history of cancer, molecular subtype, and method of detection remained associated with the prevalence of ABC after adjustment.

Finally, mammographic detection significantly reduced the prevalence of ABC compared to symptom-based diagnoses (PR = 0.40, 95 % CI 0.31‒0.51).

## Discussion

In the hospital-based cohort, asymptomatic patients diagnosed with abnormal mammogram findings had a lower prevalence of ABC after adjusting for sociodemographic covariates and molecular subtypes (PR = 0.40, 95 % CI 0.31‒0.51). Tumors detected by mammography tended to be smaller and showed a significantly lower prevalence of lymph node disease compared to symptom-based diagnoses. Additionally, the present study showed that the Luminal A subtype had the highest prevalence of mammographic detection. Farshid et al. found a similar distribution of subtypes in an organized screening context.^33^

This reduced prevalence of ABC in the screen-detected population exceeds expectations, given the 38 % risk reduction observed in a meta-analysis of clinical trials (RR = 0.62, 95 % CI 0.46–0.83)^34^ and 25 % in a meta-analysis of population-based evidence from nine Swedish counties (RR = 0.75, 95 % CI 0.66‒0.84).^35^ It is important to interpret these results with caution, given the differences in study populations, research designs, and varying levels of screening organization. Population-based cohorts and clinical trials followed healthy individuals in organized screening experiments, comparing incidence-based rates through relative risks. The hospital-based study evaluated prevalence differences only in BC-confirmed cases within the context of opportunistic mammographic screening.

In the present study, the authors found that sociodemographic factors were associated with the prevalence of mammographic detection. The authors found a reduced prevalence of mammographic detection among women below 40 (PR = 0.16, 95 % CI 0.08‒0.33) and aged 40‒49 (PR = 0.64, 95 % CI 0.50‒0.84), compared to 50‒69-year-old patients, after adjusting for family history of cancer and other covariates. The Amazona III study found similar results, with a significant difference in screen-detected BC between patients below 40 (26.6 %) and over 50 years old (37.4 %) (PR = 1.55, 95 % CI 1.21‒1.98).^23^ Researchers suggest that women above 69 years old were less likely to engage in screening practices,^18^ although this difference was non-significant in this study.

The authors found that single women had a 32 % reduced prevalence of mammographic detection after multivariate adjustment (PR = 0.68, 95 % CI 0.49‒0.94). Additionally, widows showed a significantly lower prevalence of mammographic detection, only in univariate analyses. These findings suggest that single and widowed women may experience difficulties in performing recommended preventive practices. A survey of healthy women over 40 showed that those without a partner had an increased probability of not performing yearly breast clinical examinations (PR = 1.49, 95 % CI 1.08‒2.08).^19^

In the present study, brown/black women had a lower prevalence of mammographic detection (PR = 0.68, 95 % CI 0.54‒0.86) than white women. These findings corroborate the AMAZONA III results, where white patients had a 77 % higher raw prevalence of mammographic detection.^23^ In the aforementioned survey of healthy women over 40, the prevalence of not performing mammography in the last two years, adjusted for sociodemographic covariates, was 39 % higher in non-white women (PR = 1.39, 95 % CI 1.03‒1.86).^19^

In this study, the prevalence of mammographic detection was not associated with education or family history of cancer, unlike the findings from Vieira et al.^14^ and Guerra et al.^36^ Vieira et al. observed that education was associated with prior BC screening among women in São Paulo, where the odds of never having performed a mammogram were doubled for illiterate women compared to those with more than nine years of education (OR = 2.53, 95 % CI 2.00‒3.20).^14^ Guerra et al. demonstrated in a survey that adherence to screening was higher in women with a family history of BC. Moreover, Guerra found that adherence to BC screening was slightly lower in Evangelicals (53.5 %) than Catholics (58.9 %),^36^ similar to the results in univariate regression, where evangelical women's prevalence of mammographic detection was lower than that of Catholics (PR = 0.73, 95 % CI 0.58‒0.93). These studies suggest those are vulnerable populations that should be addressed in tailored preventive strategies.

The percentage of ABC in the present sample was 47.7 %, higher than the 32 % reported in the AMAZONA III study,^28^ with differing proportions between those dependent on the public health system and those with private insurance.^23^ The elevated proportion of ABC in this study may be attributed to the study design and location, as the research was conducted in a state-reference hospital attending exclusively publicly insured populations.

In the initial analyses, the authors found an increased prevalence of ABC associated with age, marital status, education, race/color, family history, and molecular subtype. After multivariate adjustment, widowed women, women over 70 years old, women with a family history of breast/ovarian cancer, not reported race/color, and symptomatic detection of BC remained associated with increased prevalence of ABC. In univariate regression, the authors found that women between 30‒39 years old had an increased prevalence of advanced stage (PR = 1.31, 95 % CI 1.13‒1.52). The present findings align with Franzoi et al., who found that women aged below 40 had more stage III disease compared to women over 40 years old (36.8 % vs. 25.1 %).^28^

Education level was an important factor associated with the prevalence of ABC in the population. Cross-sectional evidence found ABC was 38 % higher in women with none or incomplete fundamental education compared to undergraduates (PR = 1.38, 95 % CI 1.33‒1.43). In the present study, higher education was associated with a 26 % reduction in the raw prevalence of ABC compared to the lowest level (PR = 0.74, 95 % CI 0.57‒0.96). Evidence from case-control studies showed larger differences with adjusted odds ratios of 0.32 (95 % CI 0.29–0.35)^37^ and 0.41 (95 % CI 0.38–0.44).^38^

In this study, the prevalence of ABC was 43.9 % in white women and 49.4 % in brown/black patients (p = 0.051). Interestingly, the authors found that the prevalence of ABC patients was 66 % in those not reporting their race/color, even after multivariate adjustments (PR = 1.18, 95 % CI 1.04‒1.34). Evidence from case-control designs shows larger racial differences in the prevalence of ABC; in Rio de Janeiro, brown (OR = 1.63, 95 % CI 1.01‒2.65) and black women (OR = 1.40, 95 % CI 0.95‒2.06) showed higher odds of ABC compared to white women.^24^ Two studies analyzing health records found that the odds of ABC were between 18 % to 30 % higher in brown and 45 % to 63 % higher in black women.^37^^,^^38^

In Brazil, non-white women had several demographic differences compared to white women, such as lower income, lower educational levels,^24^ and higher prevalence of triple-negative disease,^5^ impacting both mammographic detection and ABC. The literature describes unfavorable clinicopathologic features in young women with BC, such as less screen detection, higher tumor grade, more HER2 positive disease, less positive hormone receptor status, and more triple-negative subtype, increasing the prevalence of the advanced-stage disease.^28^

The main limitations of this study include the absence of standardized data on individual screening history and previous results, and recall bias may affect the quality of this information. Furthermore, sociodemographic traits such as education level and self-declared race/color exhibited a high frequency of missing data. Regarding self-reported race, concerns have been raised in the national literature.^39^ Consequently, it is important to carefully evaluate missing data on this trait, particularly in contexts with high inequality. When defining mammographic detection, the absence of symptoms may be affected by recall bias. Additionally, the absence of symptoms or signs was reported by clinicians after breast clinical examination. Therefore, the authors must carefully interpret the results, considering the study design and population setting at a state-reference hospital in the most populated state of Brazil.

## Conclusions

The Luminal A molecular subtype emerges as the most prevalent in mammographic detection. However, a concerning trend is evident among women under 50, who often receive a BC diagnosis only after symptoms manifest due to the absence of systematic screening in this age group, leading them to undergo mammography only when they perceive a need. These findings support adopting medical societies´ guidelines, lowering the starting age to 40, and shifting the mammogram schedule from biennial to annual.

Education plays a key role in developing BC risk awareness and promoting early detection, likely due to increased healthcare-seeking behavior, greater information-seeking capabilities, and higher income levels. Certain sociodemographic factors, such as race, religion, and especially education level, underscore the barriers that impact vulnerable groups' access to screening programs. These disparities highlight the need for tailored interventions and educational efforts to bridge the gaps in BC detection and treatment outcomes.

## Data availability

The data that support the findings of this study are not openly available due to reasons of sensitivity and are available from the corresponding author upon reasonable request.

## Author contributions

Fernando Wladimir Silva Rivas: Participated in the conceptualization and design of the study, data curation (data collection), formal analysis, writing the original draft and review of the final version of this manuscript.

Rodrigo Gonçalves: Participated in the conceptualization and design of the study, data curation (data analysis and interpretation), formal analysis, writing the original draft and editing and review of the final version of this manuscript.

Bruna Salani Mota: Participated in the conceptualization and design of the study and editing and review of the final version of this manuscript.

Isabel Cristina Esposito Sorpreso: Participated in the conceptualization and design of the study and editing and review of the final version of this manuscript.

Tatiana Natasha Toporcov: Participated in the conceptualization and design of the study and editing and review of the final version of this manuscript.

José Roberto Filassi: Participated in the conceptualization and design of the study and editing and review of the final version of this manuscript.

Edia di Tullio Lopes: Participated in the conceptualization and design of the study and editing and review of the final version of this manuscript.

Laura Raíssa Schio: Participated in the conceptualization and design of the study, data curation (data analysis and interpretation), and editing and review of the final version of this manuscript.

Yann-Luc Patrick Comtesse: Participated in the conceptualization and design of the study, data curation (data analysis and interpretation), and editing and review of the final version of this manuscript.

Edmund Chada Baracat: Participated in the conceptualization and design of the study and editing and review of the final version of this manuscript.

José Maria Soares Júnior: Participated in the conceptualization and design of the study and editing and review of the final version of this manuscript.

## Declaration of competing interest

The authors declare no conflicts of interest.
